# Distance learning in Italian primary and middle school children during the COVID-19 pandemic: a national survey

**DOI:** 10.1186/s12889-021-11026-x

**Published:** 2021-06-02

**Authors:** Francesca Scarpellini, Giulia Segre, Massimo Cartabia, Michele Zanetti, Rita Campi, Antonio Clavenna, Maurizio Bonati

**Affiliations:** Laboratory for Mother and Child Health, Department of Public Health, Istituto di Ricerche Farmacologiche Mario Negri IRCCS, Via Mario Negri 2, 20156 Milan, Italy

**Keywords:** Distance education, surveys and questionnaires, COVID-19, child psychology, quarantine, social isolation, children’s mental health, home learning

## Abstract

**Background:**

School closure created difficulties for parents, who were asked to care for their children and help them with schooling, while working at home. We aimed to explore the experiences in organising school for children at home and its implications on children’s psychological well-being and educational progress during the quarantine for the COVID-19 pandemic.

**Methods:**

A nationwide online survey of mothers of primary and middle school students was conducted during the COVID-19 pandemic. Demographic data and information on distance learning organisation and children’s attitudes and behavioural changes were collected.

**Results:**

2149 mothers completed the survey, with a final sample of 1601 subjects. Large differences between primary and middle school emerged: lessons were less organised and routines were more instable for the youngest, who could not pay attention for more than 20 min (28.3%) and needed breaks every 10 min (21.6%), with lower quality of learning (40.6%), increased restlessness (69.1%), and aggressiveness (33.3%). A large use of screens was reported, with an abuse in screen time in 2%. Two thirds of mothers did not approve of distance learning (72.2%) because of their role in replacing teachers (77.8%), the effort required (66%), and the great commitment required (78.3%).

**Conclusions:**

Distance learning increased educational deprivation and social inequalities, especially for the youngest children, who lost almost one year of school. The situation was even worse for children with disabilities, who were neglected by the institutions. This period should be considered as an opportunity to correct the weaknesses of our school system.

**Supplementary Information:**

The online version contains supplementary material available at 10.1186/s12889-021-11026-x.

## Background

In response to the recent coronavirus disease 2019 (COVID-19) emergency, the Italian government closed all schools on February 23rd in order to prevent the spread of the virus in its territory. In Italy, about 8.5 million students remained confined at home [1], as in other 185 countries of the world. This affected 1.56 billion children and young people: about 89.6% of the global student population [2]. Education, however, was not interrupted and continued online. In order to guarantee educational rights, great efforts were carried out by the Italian Minister of Instruction and by the teachers to plan distance learning and ensure the continuity of classrooms through web platforms, videoconference tools, and social devices. Online schooling preserved the continuity of the academic year, although this solution created some problems for families’ well-being. Evidence of the effect of distance learning on preventing a virus’ diffusion is lacking from previous pandemics [3], whereas a few evaluations were carried out for the COVID-19 pandemic [4–6]. The severe consequences of school closures were not justified by the clinical picture of Italian children [7].

In Italy 42% of minors live in a condition of overpopulation in their own house and 7% of children and adolescents are victims of great housing distress [8]. In these houses young students have difficulties finding a quiet place in which to study, follow lessons, and do homework. In Europe, children who live in families with poor economic conditions were unable to take part in distance learning because of the absence of an internet connection or technological devices such as computers, and because of a lack of support in accessing the lessons [9, 10]. This meant that a significant group of children may have been excluded not only from learning, but also from any form of socialisation with peers and with the surrounding world. The educational inequalities and deprivation were even more serious for those who suffered from a chronic pathology, whether physical or neurocognitive, that required special educational needs [11–14]. The home confinement and the absence of social contacts represented risk factors for the development of psychological distress and other negative consequences [15, 16]. Research has shown that prolonged stress, boredom, and social isolation may lead to a higher number of mental health conditions in children, such as restlessness, aggressiveness, anxiety, and depression [17–19]. The interruption of school leads youth to spend a lot of time in front of screens and to reduce social interactions and time dedicated to sports, with consequences on sleeping rhythms and eating habits [20]. It has been estimated that 12 weeks of school interruption drops test scores significantly [21]. The protracted closure of schools represents a serious risk for the psychological well-being of children and their entire households. Parents and caregivers attempted to work remotely while caring for children. Because of parents’ home office situations and lack of multiple computers, several family members, including children who had to study, needed to use the devices contemporaneously [22]. An Italian survey conducted in April involved parents of 2-14 year old children and revealed that difficulties in dealing with quarantine had consequences on the entire family’s level of stress. Parents who reported having more problems taking care of children’s learning, finding space and time for themselves, their partners, their children, or for the activities they used to do before the lockdown, were more stressed. This, in turn, led to increased psychological symptoms in children [23]. Another online survey, conducted on 3.013 adults from 24th April to 4th May in the USA, reported that more than 7 in 10 parents felt that managing distance/online learning for their children was a significant source of stress (71%) [24]. In this period numerous articles were published regarding children’s and adult’s psychological distress due to COVID-19, but little is known about problems related to school closure [22, 25, 26]. Schools are a vital source of care for young children, and that without in-person instruction; mothers have been sidelined from the labor force [27]. Similarly, the findings on another study highlighted gender differences in the contribution to childcare but also in pandemic-related altered working conditions, with mothers being more likely to work in a system-relevant occupation, to work onsite and to change their working hours compared to fathers [28]. In order to analyze the impact of school closure and other containment restrictions on various outcomes related to children and family well-being, different surveys or interviews were addressed to mothers [29, 30]. For this reason, we involved mothers of primary and middle school students in order to collect information on their experiences with home-school organisation and its difficulties, and their opinions concerning distance learning. The aim of this study was to explore the current educational situation in Italy during the COVID-19 pandemic and the impact of school closure on the educational progress and behavioural impairment of children.

## Methods

### Participants and procedures

This was a cross-sectional, observational study carried out in Italy. A dedicated website was created for the purpose of this study. An online, structured questionnaire was developed by using Wordpress, a free open-source content management system (CMS), integrated with SurveyJS (survey library and survey creator), a library to facilitate survey creation and management. Answers were restricted to close-ended fixed choices; the survey script was available for all devices. The survey began online on May 8^th^, was available for one week, until May 15^th^, and targeted mothers of children of primary and middle school (children aged 6-15 years old). In that period, positive cases in Italy were decreasing and the nation was living a particular phase of progressive re-opening. Italian families were emerging from quarantine and it was progressively possible to meet one’s own relatives again. Many activities were resuming and parents were being asked to return to work, while schools were still closed and the educational situation still hadn’t been defined. The survey was addressed only to mothers, not minors, and was motivated. Moreover, according to previous studies, participation of women in research is usually greater than that of men [27]. No validated questionnaires related to the specific problem of school closure during a pandemic emergency were found in the literature, so we created the questions ad hoc, starting from the requests of parents published in journals and magazines in that time period, as well as from mothers’ messages received via e-mail by the Laboratory for Mother and Child Health of the Mario Negri Research Institute (See Additional File 1). The authors assert that all procedures contributing to this work comply with the ethical standards of the relevant national and institutional guidelines on care and clinical research. This study has been conducted in accordance with the Helsinky Declaration. The study was approved by the ethics committee of the San Paolo hospital (2020/ST/106) of Milan, Italy. Participation was voluntary and free; no incentives were offered to complete it. Informed consent to participate in the study was obtained only from parents before accepting to take the survey. Minors were not involved in the study. Once the link of the survey was clicked on, the participants were automatically directed to information on the study and to the informed consent.

All the items of the STROBE checklist for observational studies have been met in the present report.

### Measures

The questionnaire was created in Italian and, to submit it to as many people as possible, a snowball sampling technique was used. The link to the questionnaire was sent by e-mail, WhatsApp, and other social media to the investigators’ relatives. To encourage involvement, the invitation was sent to different mailing lists of people who were in contact with the institute. Once the link was clicked on, the participants were automatically directed to information on the study and to the informed consent. After accepting to take the survey, a set of socio-demographic questions appeared, which included age, gender, occupation, education, and area of residence, followed by other questions.

The questionnaire consisted of four sections investigating:

- **Socio-demographic variables**: about the mother (nationality, age, residential area, educational level, profession, number of room in the house, support from others, such as relatives, friends, or nannies, before the quarantine) and about the children (age, gender, brothers or sisters, school grade and type of school, academic achievement, chronic disorders, and support teachers).
- **Distance learning organisation** (with or without special needs): types of tools (e.g. PC, tablet, books) adopted and frequency of use, changes in school routine, whether teachers were reachable, effort required of the child, and learning assessment.
- **Children’s attitude and behavioural changes**: level of attention during e-learning, frequency of breaks, time spent on screens, level of commitment and autonomy in keeping up with the school programme, behavioural changes (anxiety, restlessness, aggressiveness, and sleeping or mood disorders). Each symptom was rated as mild, moderate or severe.
- **Mother’s difficulties and opinion on distance learning**: difficulties in managing work tasks and home schooling, effort required and level of commitment in supporting children, distance learning implications, and future perspectives for the upcoming school re-opening.

### Statistical analysis

Data are reported as number and percentage of responders to compare the characteristics of primary school and middle school students. Data analysis was performed using frequency distributions for categorical variables, reported as number of responders, and summarized using percentages. Associations were tested using chi-square or Fisher’s exact test where applicable. Continuous variables were summarized using median and interquartile range and statistically significant differences were evaluated using two sample Wilcoxon’s test. Where data were missing, in analyses of prevalence-evaluated characteristics, we used pairwise deletion, so that all variable data were used; and in analyses of odd ratios (OR), we used listwise deletion, so that data from the same participants were used in bivariate and multivariate models, enabling comparison. Sensitivity analysis was performed by running two separate models, adding confounders with missing values. Statistical significance was evaluated using 95% confidence interval and a two-tailed *p*-value of < 0.05. All the statistical analyses were performed using SAS software, version 9.4 (SAS, Institute Inc., Cary, NC, USA).

## Results

### Socio-demographic information on mothers and children

In total, 1,601 responders completed the online survey. Of these, 70.2% were from northern Italy, and, in particular, 50.7% were from the Lombardy Region (the most represented and representative in the survey, and the one that suffered the most from the virus in Italy and in the world at the time of the survey). The mothers were 39-49 years old, had a tertiary level of education (46.6%), and were mainly workers (66%) as employees (64.7%), and were making use of the smart working option (i.e. working from home, 62.5%). The majority of mothers had children in primary school (71.7%) and reported greater difficulties in providing support for their children’s educational learning than mothers of older students (OR = 3.16, CI 2.23-4.47). Children were 7-13 years old, 1148 from primary school, and 453 from middle school. Those who attended primary school were more frequently an only child (OR = 1.70, CI 1.34–2.16). The majority of students attended public school, but private schools had a larger proportion of primary school students (OR = 1.96, CI 1.27–3.02). School performance before quarantine was, for the majority of the students, good or better (88.7%), with higher results for the primary school students and lower performance for middle school students (Table 1).

### Distance learning organisation

#### Tools

The most commonly used instruments were computer, PC, smartphone/tablet, and books, the latter especially in primary school (OR = 1.46, CI 1.16–1.83). Most (80.7%) respondents had no difficulties with the use of technology, but 1.5% was not able to use it because the instruments were not available. The frequency of PC use was lower in primary school students (OR = 3.48, CI 2.06-5.90). Both groups used web-platform tools (59.5%) such as Edmodo or Google Suites for Education, messaging tools (42.7%), WhatsApp or FaceTime, and the videoconference tools (22.9%) Zoom or Skype. The electronic register software was less used by primary school teachers (OR = 0.45, CI 0.35-0.57), which preferred to use YouTube (OR = 1.44, CI 1.14-1.83). However, 47.7% of respondents reported that none of the mentioned tools was used by the school. The frequency of web tools use was lower for primary school children (OR = 3.58, CI 2.45-5.23). Video-lessons (recorded or in streaming) were conducted mainly by middle school teachers (OR = 0.39, CI 0.27-0.56), as well as studying with books (OR = 0.52, CI 0.41-0.65). For both groups the teaching modalities were assigning homework (91.4%), watching films and documentaries (62.9%), and sending students school materials and documents (59.4%) such as slides and links**.**

#### Learning organisation

About one fourth (26.8%) of respondents considered distance learning disorganised and characterised by a different routine compared to presence in school, especially for primary school students. More specifically, primary school students were faced with more instable routines (OR = 1.50, CI 1.24-2.01) and less organisation (OR = 1.42, CI 1.09-1.85). Great effort, however, was required for studying and doing homework for both groups (81.2%).

According to 31.1% of respondents, teachers could not be contacted, although primary school teachers were more easily available than middle school teachers (OR = 0.55, CI = 0.44 – 0.70).

#### Learning assessment

Assessment was carried out in 90.5% of students and mainly consisted of teachers’ homework revision (74.3%) without attribution of grades (43.8%), in particular in primary school. In middle school, tests and oral exams were mostly planned (77.7%) and grades varied from previous school performance, with lower grades almost twice as likely in primary school students (OR = 0.49, CI 0.30-0.78). Concerning primary schools, 11.5% of students were not assessed and more than half did not receive any grades (Table 2).

**Table 1:** Socio-demographics mothers and children variables by School

	Primary school	Middle school	Total	OR	CI 95%	p-value
**Mothers**						
Age	42,5 (39,0 -46,0)	46,0 (43,0- 49,0)	44,0 (39–49)			< 0.0001
**Region**						
North	775 (68.1)	337 (75.6)	1.112 (70.2)	1.00	(Ref.)	0.0021
Centre	214 (18.8)	52 (11.7)	266 (16.8)	1.79	(1.29–2.49)	
South	149 (13.1)	57 (12.8)	206 (13.0)	1.14	(0.82–1.58)	
Missing	10	7	17			
**Education**						
First level	187 (17.5)	95 (21.7)	282 (18.7)	0.64	(0.48–0.87)	0.0019
Second level	352 (33.0)	170 (38.8)	522 (34.7)	0.68	(0.53–0.87)	
Tertiary level	528 (49.5)	173 (39.5)	701 (46.6)	1.00	(Ref.)	
Missing	81	15	96			
**Actually employed**						
Yes	684 (65.7)	286 (66.7)	970 (66.0)	1.00	(Ref.)	0.7238
No	357 (34.3)	143 (33.3)	500 (34.0)	1.04	(0.82–1.32)	
Missing	107	24	131			
**Work**						
Employers	724 (65.2)	277 (63.5)	1.001 (64.7)	1.00	(Ref.)	0.5101
Freelance	203 (18.3)	78 (17.9)	281 (18.2)	1.00	(0.74–1.34)	
Housewives	128 (11.5)	62 (14.2)	190 (12.3)	0.79	(0.57–1.10)	
Unemployed	56 (5.0)	19 (4.4)	75 (4.8)			
Missing	37	17	54			
**Smart working**						
Yes	408 (61.5)	181 (64.9)	589 (62.5)	1.00	(Ref.)	0.3342
No	255 (38.5)	98 (35.1)	353 (37.5)	1.15	(0.86–1.54)	
Missing	21	7	28			
**Difficulties balance work/child**						
Yes	578 (87.8)	192 (69.6)	770 (82.4)	3.16	(2.23–4.47)	< 0.0001
No	80 (12.2)	84 (30.4)	164 (17.6)	1.00	(Ref.)	
Missing	26	10	36			
**Children**	1148	453	1601			
Age	8,0 (7,0 - 9,0)	12,0 (11,0 - 13,0)	9,0 (7,0 - 11,0)			< 0.0001
**Gender**						
Female	545 (48.2)	213 (47.7)	758 (48.1)	0.98	(0.78–1.22)	0.8357
Male	585 (51.8)	234 (52.3)	819 (51.9)	1.00	(Ref.)	
Missing	18	6	24			
**Brothers/Sisters**						
Yes	637 (57.9)	307 (70.1)	944 (61.4)	1.00	(Ref.)	< 0.0001
No	463 (42.1)	131 (29.9)	594 (38.6)	1.70	(1.34–2.16)	
Missing	48	15	63			
**Type of School**						
Public	917 (88.8)	418 (93.9)	1.335 (90.3)	1.00	(Ref.)	0.0021
Private	116 (11.2)	27 (6.1)	143 (9.7)	1.96	(1.27–3.02)	
Missing	115	8	123			
**School performance**						
No sufficient	11 (1.0)	8 (1.8)	19 (1.2)	0.48	(0.19–1.21)	< 0.0001
Sufficient	94 (8.4)	65 (14.4)	159 (10.2)	0.50	(0.34–0.73)	
Good	305 (27.3)	180 (40.0)	485 (31.0)	0.59	(0.45–0.77)	
Very good	378 (33.9)	131 (29.1)	509 (32.5)	1.00	(Ref.)	
Excellent	328 (29.4)	66 (14.7)	394 (25.2)	1.72	(1.24–2.40)	
Missing	32	3	35			
Total	1148	435	1601

**Table 2:** Distance Learning organisation by School

	Primary school	Middle school	Total	OR	CI 95%	p-value
**Instruments (flag)**						
Computer	130 (11.5)	70 (15.6)	200 (12.6)	0.70	(0.51–0.96)	0.0275
PC	727 (64.2)	320 (71.1)	1.047 (66.1)	0.73	(0.57–0.92)	0.0084
Smartphone/Tablet	666 (58.8)	277 (61.6)	943 (59.6)	0.89	(0.71–1.11)	0.3105
Books	800 (70.6)	280 (62.2)	1.080 (68.2)	1.46	(1.16–1.83)	0.0012
Missing	15	3	18			
**Difficulties with technologies**						
Not used	17 (1.5)	7 (1.6)	24 (1.5)	0.90	(0.37–2.18)	0.0579
Some	184 (16.3)	95 (21.4)	279 (17.7)	0.71	(0.54–0.94)	
None	927 (82.2)	342 (77.0)	1.269 (80.7)	1.00	(Ref.)	
Missing	20	9	29			
**Frequency of PC use**						
Low	112 (10.2)	17 (3.8)	129 (8.3)	3.48	(2.06–5.90)	< 0.0001
Moderate	355 (32.3)	95 (21.3)	450 (29.1)	1.97	(1.52–2.57)	
Often	632 (57.5)	334 (74.9)	966 (62.5)	1.00	(Ref.)	
Missing	12	3	15			
**Type of tools (flag)**						
Edmodo, Google Suits for Education	678 (59.8)	265 (58.8)	943 (59.5)	1.04	(0.84–1.30)	0.7062
WhatsApp/FaceTime	458 (40.4)	219 (48.6)	677 (42.7)	0.72	(0.58–0.89)	0.0030
Zoom, Skype	273 (24.1)	90 (20.0)	363 (22.9)	1.27	(0.97–1.66)	0.0783
Electronic register	628 (55.4)	331 (73.4)	959 (60.5)	0.45	(0.35–0.57)	< 0.0001
YouTube	427 (37.7)	133 (29.5)	560 (35.3)	1.44	(1.14–1.83)	0.0022
None	496 (43.7)	260 (57.6)	756 (47.7)	0.57	(0.46–0.71)	< 0.0001
Missing						
**Frequency of web tools use**						
Low	199 (28.6)	38 (10.2)	237 (22.1)	3.58	(2.45–5.23)	< 0.0001
Moderate	287 (41.2)	97 (26.0)	384 (35.9)	2.02	(1.53–2.67)	
Often	401 (57.5)	274 (73.5)	675 (63.1)	1.00	(Ref.)	
Missing	9	2	11			
**Teaching modalities (flag)**						
Homework	1.044 (91.0)	416 (92.4)	1.460 (91.4)	0.83	(0.55–1.24)	0.3605
Film/ Documentaries	718 (62.6)	287 (63.8)	1.005 (62.9)	0.95	(0.76–1.19)	0.6606
School Material	686 (59.8)	263 (58.4)	949 (59.4)	1.06	(0.85–1.32)	0.6176
Video-lessons	936 (81.6)	414 (92.0)	1.350 (84.5)	0.39	(0.27–0.56)	< 0.0001
Books	663 (57.8)	327 (72.7)	990 (62.0)	0.52	(0.41–0.65)	< 0.0001
Missing	1	3	4			
**Distance learning organisation**						
Yes	766 (71.3)	335 (77.9)	1.101 (73.2)	1.00	(Ref.)	0.0092
No	308 (28.7)	95 (22.1)	403 (26.8)	1.42	(1.09–1.85)	
Missing	74	23	97			
**Stable routine**						
Yes	271 (24.7)	149 (34.2)	420 (27.4)	1.00	(Ref.)	0.0002
No	826 (75.3)	287 (65.8)	1.113 (72.6)	1.50	(1.24–2.01)	
Missing	51	17	68			
**Child effort**						
Yes	899 (81.7)	352 (80.0)	1.251 (81.2)	1.00	(Ref.)	0.4329
No	201 (18.3)	88 (20.0)	289 (18.8)	0.89	(0.68–1.18)	
Missing	48	13	61			
**Teachers reachability**						
Yes	786 (72.6)	256 (59.4)	1.042 (68.9)	1.00	(Ref.)	< 0.0001
No	296 (27.4)	175 (40.6)	471 (31.1)	0.55	(0.44–0.70)	
Missing	66	22	88			
**Assessment**						
Yes	993 (88.5)	432 (95.6)	1.425 (90.5)	1.00	(Ref.)	< 0.0001
No	129 (11.5)	20 (4.4)	149 (9.5)	2.81	(1.73–4.55)	
Missing	26	1	27			
**Homework revision**						
Teachers	846 (75.1)	321 (72.3)	1.167 (74.3)	1.00	(Ref.)	0.0449
Self-revision	65 (5.8)	17 (3.8)	82 (5.2)	1.45	(0.84–2.51)	
Both	215 (19.1)	106 (23.9)	321 (20.4)	0.77	(0.59–1.00)	
Missing	22	9	31			
**Grades attribution**						
Yes	517 (46.9)	343 (80.0)	860 (56.2)	1.00	(Ref.)	< 0.0001
No	585 (53.1)	86 (20.0)	671 (43.8)	4.51	(3.46–5.88)	
Missing	46	24	70			
**Planned assessment**						
Yes	558 (59.3)	327 (77.7)	885 (65.0)	1.00	(Ref.)	< 0.0001
No	383 (40.7)	94 (22.3)	477 (35.0)	2.39	(1.83–3.11)	
Missing	52	11	63			
**Grades variability**						
Higher	62 (12.5)	53 (16.2)	115 (14.0)	0.68	(0.46–1.02)	0.0037
Same	398 (80.4)	232 (70.9)	630 (76.6)	1.00	(Ref.)	
Lower	35 (7.1)	42 (12.8)	77 (9.4)	0.49	(0.30–0.78)	
Missing	22	16	38

**Table 3:** Children attitude and behaviour by School

	Primary school	Middle school	Total	OR	CI 95%	p-value
**Attention span**						
≤ 20 min	317 (28.3)	60 (13.6)	377 (24.2)	2.39	(1.75–3.25)	< 0.0001
20 min- 1 h	673 (60.1)	304 (68.9)	977 (62.6)	1.00	(Ref.)	
> 1 h	129 (11.5)	77 (17.5)	206 (13.2)	0.76	(0.55–1.03)	
Missing	29	12	41			
**Breaks frequency**						
Every 10 min	239 (21.6)	37 (8.4)	276 (17.9)	2.25	(1.53–3.30)	< 0.0001
Every 20–30 min	523 (47.2)	182 (41.6)	705 (45.6)	1.00	(Ref.)	
Every 1 h	345 (31.2)	219 (50.0)	564 (36.5)	0.55	(0.43–0.70)	
Missing	41	15	56			
**Restlessness during distance learning**						
Yes	534 (48.3)	179 (40.5)	713 (46.1)	1.37	(1.10–1.72)	0.0055
No	572 (51.7)	263 (59.5)	835 (53.9)	1.00	(Ref.)	
Missing	42	11	53			
**Distance learning duration**						
≤ 2 h	737 (65.5)	89 (19.8)	826 (52.5)	1.00	(Ref.)	< 0.0001
2–4 h	340 (30.2)	267 (59.5)	607 (38.6)	0.15	(0.12–0.20)	
4–6 h	48 (4.3)	93 (20.7)	141 (9.0)	0.06	(0.04–0.09)	
Missing	23	4	27			
**Screen time other than distance learning**						
≤ 2 h	660 (66.8)	133 (33.8)	793 (57.4)	1.00	(Ref.)	< 0.0001
2–4 h	302 (30.6)	201 (51.1)	503 (36.4)	0.30	(0.23–0.39)	
4–6 h	26 (2.6)	59 (15.0)	85 (6.2)	0.09	(0.05–0.15)	
Missing	160	60	220			
**Internet activities (flag)**						
Videogames	463 (43.2)	264 (59.1)	727 (47.9)	0.53	(0.42–0.66)	< 0.0001
Tutorial	387 (36.1)	197 (44.1)	584 (38.5)	0.72	(0.57–0.90)	0.0038
Film/ TV series	778 (72.6)	303 (67.8)	1.081 (71.2)		1.26 (0.99–1.60)	0.0568
Social	122 (11.4)	201 (45.0)	323 (21.3)	0.16	(0.12–0.21)	< 0.0001
Study	310 (28.9)	174 (38.9)	484 (31.9)	0.64	(0.51–0.81)	0.0001
Missing	77	6	83			
**Behaviour changes**						
Yes	692 (62.5)	241 (54.5)	933 (60.2)	1.39	(1.11–1.73)	0.0040
No	416 (37.5)	201 (45.5)	617 (39.8)	1.00	(Ref.)	
Missing	40	11	51			
**Symptoms (flag)**						
Restlessness	459 (69.1)	129 (56.6)	588 (65.9)	1.72	(1.26–2.34)	0.0006
Aggressiveness	221 (33.3)	57 (25.0)	278 (31.2)	1.50	(1.06–2.10)	0.0198
Anxiety	176 (26.5)	78 (34.2)	254 (28.5)	0.69	(0.50–0.96)	0.0261
Sleeping rhythm	270 (40.7)	96 (42.1)	366 (41.0)	0.94	(0.69–1.28)	0.7024
Mood lability	107 (16.1)	43 (18.9)	150 (16.8)	0.83	(0.56–1.22)	0.3390
Missing	28	13	41			
**Restlessness**						
No	649 (59.3)	313 (71.0)	962 (62.7)	1.00	(Ref)	< 0.0001
Mild	103 (9.4)	45 (10.2)	148 (9.6)	1.10	(0.76–1.61)	
Moderate	274 (25.0)	72 (16.3)	346 (22.5)	1.84	(1.37–2.46)	
Severe	68 (6.2)	11 (2.5)	79 (5.1)	1.50	(1.06–2.10)	
Missing	54	12	66			
**Aggressiveness**						
No	887 (81.8)	385 (87.7)	1.272 (83.5)	1.00	(Ref)	0.0419
Mild	41 (3.8)	9 (2.1)	50 (3.3)	1.98	(0.95–4.1)	
Moderate	117 (10.8)	34 (7.7)	151 (9.9)	1.49	(1.00–2.23)	
Severe	39 (3.6)	11 (2.5)	50 (3.3)	1.54	(0.78–3.04)	
Missing	64	14	78			
**Anxiety**						
No	932 (84.3)	364 (82.4)	1.296 (83.7)	1.00	(Ref)	0.4920
Mild	52 (4.7)	24 (5.4)	76 (4.9)	0.85	(0.51–1.39)	
Moderate	89 (8.0)	44 (10.0)	133 (8.6)	0.79	(0.54–1.16)	
Severe	33 (3.0)	10 (2.3)	43 (2.8)	1.29	(0.63–2.64)	
Missing	42	11	53			
**Sleeping rhythm**						
No	838 (76.3)	346 (79.0)	1184 (77.1)	1.00	(Ref)	0.1512
Mild	62 (5.6)	13 (3.0)	75 (4.9)	1.97	(1.07–3.63)	
Moderate	112 (10.2)	48 (11.0)	160 (10.4)	0.96	(0.67–1.38)	
Severe	86 (7.8)	31 (7.1)	117 (7.6)	1.15	(0.75–1.76)	
Missing	50	15	65			
**Mood lability**						
No	1001 (91.0)	399 (90.5)	1.400 (90.9)	1.00	(Ref)	0.3359
Mild	38 (3.5)	10 (2.3)	48 (3.1)	1.51	(0.75–3.07)	
Moderate	42 (3.8)	24 (5.4)	66 (4.3)	0.70	(0.42–1.17)	
Severe	19 (1.7)	8 (1.8)	27 (1.8)	0.95	(0.41–2.18)	
Missing	48	12	60

**Table 4:** Mothers’ opinion about Distance learning by School

	Primary school	Middle school	Total	OR	CI 95%	p-value
**Mother effort**						
Yes	798 (73.5)	199 (46.8)	997 (66.0)	3.15	(2.50–3.98)	< 0.0001
No	287 (26.5)	226 (53.2)	513 (34.0)	1.00	(Ref.)	
Missing	63	28	91			
**Mother commitment**						
Yes	896 (82.5)	300 (68.0)	1.196 (78.3)	2.21	(1.71–2.85)	
No	190 (17.5)	141 (32.0)	331 (21.7)	1.00	(Ref.)	< 0.0001
Missing	62	12	74			
**Replacing teachers**						
Yes	936 (85.0)	260 (59.5)	1.196 (77.8)	3.86	(2.99–4.97)	
No	165 (15.0)	177 (40.5)	342 (22.2)	1.00	(Ref.)	< 0.0001
Missing	47	16	63			
**Difficulties with (flag)**						
Organisation	541 (49.7)	212 (51.0)	753 (50.1)	0.87	(0.64–1.17)	0.3526
Time dedicated	895 (82.3)	223 (53.6)	1.118 (74.3)	3.53	(2.54–4.88)	< 0.0001
Technologies	523 (48.1)	266 (63.9)	789 (52.5)	0.52	(0.38–0.71)	< 0.0001
Missing	60	37	97			
**Child autonomy**						
Yes	205 (18.1)	214 (47.9)	419 (26.6)	1.00	(Ref.)	< 0.0001
No	925 (81.9)	233 (52.1)	1.158 (73.4)	4.14	(3.26–5.269	
Missing	18	6	24			
**Child commitment**						
Yes	458 (41.1)	276 (63.3)	734 (47.3)	1.00	(Ref.)	< 0.0001
No	657 (58.9)	160 (36.7)	817 (52.7)	0.40	(0.32–0.51)	
Missing	33	17	50			
**Scarce learning**						
Yes	455 (40.6)	130 (29.5)	585 (37.4)	1.63	(1.29–2.07)	< 0.0001
No	667 (59.4)	311 (70.5)	978 (62.6)	1.00	(Ref.)	
Missing	26	12	38			
**Distance learning in the future**						
Yes	262 (23.8)	165 (37.9)	427 (27.8)	1.00	(Ref.)	< 0.0001
No	838 (76.2)	270 (62.1)	1.108 (72.2)	1.95	(1.54–2.48)	
Missing	48	18	66			
**Child care pre COVID-19 (flag)**						
Parents	821 (72.5)	333 (74.7)	1.154 (73.1)	0.90	(0.70–1.15)	0.3885
Grandparents	541 (47.8)	178 (39.9)	719 (45.6)	1.38	(1.10–1.72)	0.0046
Others	230 (20.3)	74 (16.6)	304 (19.3)	1.28	(0.96–1.71)	0.0910
Missing	16	7	23			
**Child care post COVID-19 (flag)**						
Parents	963 (84.9)	391 (87.9)	1.354 (85.8)	0.78	(0.56–1.08)	0.1321
Grandparents	361 (31.8)	94 (21.1)	455 (28.8)	1.74	(1.35–2.26)	< 0.0001
Others	164 (14.5)	53 (11.9)	217 (13.7)	1.25	(0.90–1.74)	0.1852
Missing	14	8	22			

### Children’s attitude and behavioural changes

Children’s attitude towards distance learning differed between the youngest and oldest. Primary school students could not pay attention for more than 20 minutes (OR = 2.39, CI 1.75-3.25), needed breaks every 10 minutes (OR = 2.25, CI 1.53-3.30), and presented more restlessness during video lessons (OR = 1.37, CI 1.10-1.72). Results also revealed a large use of screens (minimum 2 hours of video lessons per day for more than half of students). This was especially true for middle school students, who spent several hours in front of a screen, considering both distance learning and screen time other than distance learning compared to primary school students. In 2% of the students there was an abuse of media use, with 8 -12 hours of screen time. Primary school students spent less time on internet activities such as videogames (OR = 0.53, CI 0.42-0.66) and social networks (OR = 0.16, CI 0.12-0.21) than middle school students.

A majority (60.2%) of mothers observed behavioural changes in their children, in particular in the youngest (OR = 1.39, CI 1.11-1.73). The most frequently observed symptoms were restlessness (69.1%) and aggressiveness (33.3%) in the youngest, and anxiety (34.2%) in the oldest. No differences emerged between subjects concerning sleeping rhythm and mood lability. The level of restlessness and aggressiveness was particularly severe for primary school children (OR = 1.72, CI 1.26 – 2.44; OR = 1.50, CI 1.06 – 2.10) compared to middle school children (Table 3).

#### Special needs students

In all, 5.5% of our sample suffered from a chronic disorder, such as a medical (25.6%) or physical condition (11.5%) or neurodevelopmental disorder (66.7%). Children with a support teacher (5.9%) attended lessons online only once a week (53.7%). Compensatory measures consisted mainly in concept maps (51%), additional time (39%), and reduced tasks (33.5%). In all, 14.7% of the children did not receive any support. The main teaching mode was via video lessons (77.4%), and no differences emerged between primary and middle school students.

### Mothers’ difficulties and opinions on distance learning

Compared to middle school, mothers of primary school students expressed worse opinions about distance learning. They reported greater effort (OR = 3.15, CI 2.50 – 3.98) and higher need for commitment (OR = 2.21, CI 1.71 – 2.85) in supporting their children, and sometimes in replacing teachers (OR 3.86, CI 2.99- 4.97), for example when their child did not know how to solve a math problem. Half of the mothers were faced with difficulties in daily organisation and 82.3% reported not having enough time for their children and the whole family (OR = 3.53, CI 2.54-4.88). Primary school children were less independent (OR = 4.14, CI 3.26-5.27), with low levels of learning (OR = 1.63, CI 1.29-2.07). Mothers rejected distance learning for the future (72.2%), in particular for primary school students (OR = 1.95, CI 1.54-2.48). Before COVID-19, 45% of mothers received help from grandparents in taking care of their children, while only 28.8% could rely on grandparents in the future, in particular for middle school children (OR = 1.74, CI 1.35-2.26) (Table 4).

## Discussion

COVID-19 has forced the government to take drastic preventive actions, such as the quarantine and school closure. Low transmission of SARS-CoV-2 within schools, at least among younger students was reported. However, entire schools are frequently closed in the fear of larger outbreaks [32]. Our research explored the current educational situation in one of the most affected countries in the world and how distance learning had been organised in response to school closure. Home confinement was seen as a limitation of freedom for children, who were forced to drastically change their habits, while school closure deprived children of socialisation opportunities, with negative consequences on levels of autonomy and independence. Schools have always been at the heart of the rights of children and adolescents and their families [33]. School is, indeed, a place where children can pursue new interests, build relationships, confront themselves with peers, grow up and become adults. This survey highlighted negative effects of distance learning on children’s attitude and behaviour and found that, in our sample, distance learning was not well regarded by parents because of the lack of organisation and planned routine, and because of the absence of assessment of the children’s work and the difficulty in reaching the teachers. One of the most frightening data highlighted by our research is that 1.5% of our sample did not participate in distance learning because they did not have access to technological tools. This result is in line with a global analysis of the potential reach of remote learning policies conducted by UNICEF in 33 countries. Globally, at least 31 percent of students from pre-primary to upper secondary schools cannot be reached due to either a lack of policies supporting digital and broadcast remote learning or a lack of the household assets needed to receive digital or broadcast instruction [30].

Our results support previous research focused on the importance of school and social interactions for the well-being of children and their role in preventing the development of psychological distress [15] and other mental health conditions such as anxiety and depression [17, 31]. The lack of structured, daily school life and the absence of interactions with peers, together with an instable quarantine routine, had an impact on the emotional and behavioural conditions of children. In other surveys mothers reported behavioural changes in their children during lockdown, such as an increment in restlessness and aggressiveness, boredom, sadness, attention deficit, hyperactivity, and regressive behaviours [32, 33]. Mothers also reported difficulties in motivating their child to study [24].

These behavioural and emotional impairments were greater in primary school students than in middle school students: the instable and badly structured distance learning for the youngest led to increased levels of restlessness and aggressiveness, little commitment during lessons, and scarce autonomy. In this unusual situation, parents were asked to support their children, in particular the youngest, in the educational process and, at the same time, to work and provide home care, with negative consequences on their own distress level [24]. Some mothers reported not having enough time to help their child with schoolwork, while grandparents and family friends were not able to help due to the lockdown [22]. According to the literature [9, 33, 34], families with two or more children and living in low socio-economic conditions were penalised because of lack of space, time dedicated to children, and difficulties with technologies [35–38]. Quarantine has increased the gap between families with high and low socio-economic levels, increased differences, and destroyed the concept of equal opportunity. This situation not only produced social inequalities, but also educational inequalities, and did so to an even greater extent in those suffering from a chronic disorder or a disability. These children did not receive adequate support [11]. Moreover, distance learning deleted social interaction and neglected their special needs.

### Strengths and limitations

There are strengths and limitations to our study. The web data collection has considerable potential, even if the large sample of participants is not representative of the population of Italian mothers. Most participants (50.7%) were mothers from the Lombardy Region, employed, with two or more sons. Characteristics of responders may be correlated with their perceived difficulty as well as their decision to participate in the study. Moreover, childrens’ attitudes and behavioural changes have been described from maternal perspective only: this could be considered a potential bias because, as mentioned above, mothers were the ones most overwhelmed by this pandemic situation. In the future it could be useful to examine effects on children from both parents’ perspectives and to analyse their potential correlation. High-functioning users of social media who are already engaged in similar initiatives might be over-represented. On the other hand, families without technological tools or internet connection may be faced with the impossibility to participate in the study. Another limitation is the lack of inclusion of minorities and more fragile populations on several point of views, including on an economic and social point of view. Lastly, the use of cross-sectional self-reported data, as in the present study, precludes attribution of causality. The findings reflect important associations among the variables we studied, and strong corroboration between these findings and existing literature about children’s educational needs, suggesting the need for future longitudinal studies in this area.

## Conclusions

Despite the efforts provided by teachers, distance learning turned out to be useless and ineffective in replacing physical presence in school; low levels of learning, insufficient cognitive stimulation, and absence of social interactions created a gap that will be hard to fill, especially for young children, who have lost almost one year of school. One year of failure to learn may have severe repercussions on students’ cognitive, emotional, and relational capacities**.** If students do not have adequate home access to an internet device that is appropriate for learning activities, or if online learning is otherwise ineffective for them, their academic progress may be at risk. Although the extent to which students will be affected is unknown at this time, a Statistics Canada study found that students who received less instructional time because they were born just after the school entry cut-off date performed more poorly in standardized tests in reading, mathematics, and science [35, 39].

The current study is important because it highlighted the importance of school for the future of our children and its capacity to guarantee children’s educational rights and psychological well-being. The results of this research could be considered as a starting point for thinking about more supportive modalities for school and family, so that both will continue to be a point of reference for children. Investments in education are needed in order to provide a better school system, because school is more than just learning, school is a right.

## Supplementary Information

**Additional file 1.** Questionnaire used for the survey.

## Data Availability

The datasets used and/or analysed during the current study are available from the corresponding author on reasonable request.
