# Supplementary material for: Distance learning in Italian primary and middle school children during the COVID-19 pandemic: a national survey
Source: BMC Public Health. 2021 Jun 2;21:1035. doi: 10.1186/s12889-021-11026-x (PMC8170444; doi:10.1186/s12889-021-11026-x)
Supplement: Supplementary file 1 — Additional file 1. Questionnaire used for the survey. [file 12889_2021_11026_MOESM1_ESM.docx]

**Additional File 1. Questionnaire used for the survey**

| **Mothers information** | |
| --- | --- |
| 1. ***Age ___*** | |
| 1. ***District of residence ___*** | |
| 1. ***City of residence ___*** | |
| 1. ***Level of Education*** | |
| - *First level* - *Second level* - *Tertiary level* | |
| 1. ***Actually employed*** | |
| - *Yes* - *No* | |
| 1. ***Type of occupation*** | |
| - *Employers* - *Freelance* - *Housewives* - *Unemployed* | |
| 1. ***Are you working in smart working modality?*** | |
| - *Yes* - *No* | |
| 1. ***Are you facing with difficulties in balancing work and child care?*** | |
| - *Yes* | |
| - *No* | |
| 1. ***Before school closure, who took care about your child after school?*** | |
| - *Me or my husband* - *Grandparents* - *Other/Baby sitter* | |
| 1. ***Once school reopens, who will take care about your child after school?*** | |
| - *Me or my husband* - *Grandparents* - *Other/Baby sitter* | |
| **Children information** | |
| 1. ***Age ___*** | |
| 1. ***Gender*** | |
| - *Female* - *Male* | |
| 1. ***Presence of brothers or/and sisters?*** | |
| - *Yes* - *No* | |
| 1. ***Children school***  - *Primary school* - *Middle school* | |
| 1. ***Type of School*** | |
| - *Public* - *Private* | |
| 1. ***Children school performance before school closure*** | |
| - *No sufficient* - *Sufficient* - *Good* - *Very good* - *Excellent* | |
| 1. ***Does the child suffer from a chronic disorder?***  - *Yes* - *No* | |
| 1. ***What kind of disorder does your child suffer? (multiple choice)*** | |
| - *Physical condition (for example motor disability)* - *Neurodevelopmental disorder (for example ADHD, DOP, DSA)* - *Medical condition (for example Diabetes/ Epilepsy)* | |
| 1. ***Does your child need a support teacher?***  - *Yes* - *No* | |
| 1. ***Does your child is followed by a support teacher during school closure?***  - *Yes* - *No* | |
| 1. ***Which supportive teaching modalities are put in place?(multiple choice)*** | |
| - *Concept maps* - *Addition time* - *Reduced tasks* - *Exemption* | |
| 1. ***How many times are online supportive lessons put in place?*** | |
| - *Daily* - *Once a week* - *Less than once a week* | |
| **Information about distance learning organisation** | |
| 1. ***What kinds of instruments are used for distance learning? (multiple choice)*** | |
| - *Computer* - *PC* - *Smartphone/Tablet* - *Books* | |
| 1. ***Do you have difficulties with technologies?*** | |
| - *Not used because of lack of tool or internet connection* - *Some difficulties* - *No difficulties* | |
| 1. ***Frequency of PC use (only where the subject have flagged the PC option)*** | |
| - *Low* - *Moderate* - *Often* | |
| 1. ***Which type of tools are used for distance learning (multiple choice)*** | |
| - *Edmodo, Google Suits for Education* - *WhatsApp/FaceTime* - *Zoom, Skype* - *Electronic register* - *YouTube* - *None* | |
| 1. ***Frequency of web tools use (only where the subject have previously flagged the first and/or the third option)*** | |
| - *Low* - *Moderate* - *Often* | |
| 1. ***Which are the main teaching modalities (multiple choice)?*** | |
| - *Homework* - *Film/ Documentaries* - *School Material (slides, link)* - *Video-lessons* - *Books* | |
| 1. ***Is the distance learning organised?*** | |
| - *Yes, the materials and teaching modalities are stable and well organised* - *No, the materials and teaching modalities are instable and bad organised* | |
| 1. ***Is there a routine stable and organised?*** | |
| - *Yes, the lessons and the schedule are stable and organised* - *No, the lessons and the schedule are variable and disorganised* | |
| 1. ***What do you think about the workload required to the child by the school?*** | |
| - *The workload require much effort* - *The workload doesn’t require much effort* | |
| 1. ***Are the teachers reachable?*** | |
| - *Yes, they are available and helpful* - *No, it’s impossible to get in touch with them* | |
| 1. ***Do the teachers assess the children?*** | |
| - *Yes, teachers assess the children (for example with oral exams or homework revision)* - *No, the assessment is interrupted* | |
| 1. ***Homework revision is conducted by:*** | |
| - *Teachers* - *Self-revision tools are provided* - *Both* | |
| 1. ***Do the teachers assign grades?*** | |
| - *Yes* - *No* | |
| 1. ***Is the assessment planned?*** | |
| - *Yes* - *No* | |
| 1. ***Do your child grades vary compare to school in presence?*** | |
| - *Yes, grades are higher* - *No, grades are the same* - *Yes, grades are lower* | |
| **Children’s attitude and behavioural changes** | |
| 1. ***How many times does your child can pay attention during distance learning?*** | |
| - *≤20 minutes* - *Between 20 min- 1 hour* - *>1 hour* | |
| 1. ***How frequently does he/she need breaks?*** | |
| - *Every 10 minutes* - *Every 20-30 min* - *Every 1 hour* | |
| 1. ***Does your child is restless during distance learning*** | |
| - *Yes* - *No* | |
| 1. ***What is your child screen time for distance learning?*** | |
| - *≤ 2 hours* - *2-4 hours* - *4-6 hours* | |
| 1. ***What is your child leisure screen time, excluding hours of online lessons?*** | |
| - *≤ 2 hours* - *2-4 hours* - *4-6 hours* | |
| 1. ***What are the main internet activities? (multiple choice)*** | |
| - *Videogames* - *Tutorials (for example YouTube)* - *Films/ TV series* - *Social Networks (for examples Facebook, Instagram, Tik Tok)* - *Study* | |
| 1. ***Have you observed behaviour or emotional changes during school closure?*** | |
| - *Yes* - *No* |  |
| 1. ***What kind of changes have you observed? (multiple choice)*** | |
| - *Restlessness* - *Aggressiveness* - *Anxiety* - *Sleeping rhythm* - *Mood lability* | |
| 1. ***In which measure are these symptoms present? (for each one)*** | |
| - *Mild* - *Moderate* - *Severe* | |
| **Mothers’ opinion about distance learning** | |
| 1. ***Does the effort required is great?*** | |
| - *Yes* - *No* | |
| 1. ***Do you feel more committed about your child distance learning organisation?*** | |
| - *Yes* - *No* | |
| 1. ***Do you have to replace teachers, some time?*** | |
| - *Yes* - *No* | |
| 1. ***What are the main difficulties with distance learning? (multiple choice)*** | |
| - *School and routine organisation* - *Time dedicated in supporting child distance learning* - *Difficulties with technologies* | |
| 1. ***Is your child independent concerning distance learning?*** | |
| - *Yes, he/she does homework and follow video lessons by him/her self* - *No, he/she needs my help in doing homework and following video lessons* | |
| 1. ***Is your child committed with distance learning?*** | |
| - *Yes* - *No* | |
| 1. ***Do you think that the level of learning is scarce with this modality?*** | |
| - *Yes* - *No* | |
| 1. ***Do you want distance learning in the future?*** | |
| - *Yes* - *No* | |
